# Findings from the expert-novice paradigm on differential response behavior among multiple-choice items of a pedagogical content knowledge test – implications for test development

**DOI:** 10.3389/fpsyg.2023.1240120

**Published:** 2023-10-18

**Authors:** Tobias Lieberei, Virginia Deborah Elaine Welter, Leroy Großmann, Moritz Krell

**Affiliations:** ^1^IPN–Leibniz Institute for Science and Mathematics Education, Biology Education, Kiel, Germany; ^2^Freie Universität Berlin, Biology Education, Berlin, Germany

**Keywords:** expert-novice paradigm, test instrument, pedagogical content knowledge (PCK), scientific reasoning, pre-service science teachers

## Abstract

Pedagogical content knowledge (PCK) is one core dimension of teachers’ professional knowledge and comprises knowledge about conceptual ideas of learners and appropriate instructions. However, several challenges regarding the assessment of PCK are discussed in the literature: For example, PCK is a topic-specific construct and contains differentiable subdomains, which must be considered during test development. In addition, the choice of test type needs to be considered. While open-ended instruments can capture a broader range of cognitions, they often require a high level of interpretation; in contrast, multiple-choice instruments have advantages in terms of objectivity and test economy. Some challenges of assessing PCK are particularly related to multiple-choice instruments, such as an insufficient focus on specific components or the accidental assessment of teachers’ beliefs instead of PCK. To better understand and explain these challenges in developing multiple-choice PCK instruments, we exemparly used an instrument to assess PCK about scientific reasoning and considered the assumptions of the expert-novice paradigm to analyze differential response behavior between *n* = 10 researchers in the field of biology education (experts) and *n* = 10 undergraduate pre-service biology teachers (novices). As expected, experts scored significantly higher than novices. At the same time, experts answered the items more consistently than novices, i.e., showed less variance. However, the difference found was statistically insignificant. Regarding the explanations for choosing a response option, experts more often correctly identified the quintessence of the items, which means that they more often understand the items as intended and argued based on their PCK. On the other hand, novices focused more on surface characteristics, i.e., they argued rather with surface knowledge like intuition or personal experience, than choosing the response option based on their PCK. These crucial differences in how experts and novices understand the items of the used PCK instrument and how they respond based on their understanding affect different test characteristics. In conclusion, we recommend ensuring that instruments address only a few, specific PCK aspects, considering the target group of a test, and take into account that target groups with larger variability among their responses require a higher number of items to achieve satisfactory discrimination and reliability indices.

## 1. Introduction

Teachers’ professional competence has a crucial influence on the effectiveness of their teaching (Hattie, 2008; Blömeke et al., 2022). Besides motivational orientations, beliefs, values, goals, and self-regulation, professional knowledge represents a central aspect of this professional competence (Baumert and Kunter, 2013). Following Shulman (1986, 1987), professional knowledge comprises several domains, of which the most prominent include (1) pedagogical knowledge (PK; knowledge about basic principles of education, teaching, learning, and the learners), (2) content knowledge (CK; knowledge about the discipline to be taught), and (3) pedagogical content knowledge (PCK; an “amalgam” of CK and PK, including knowledge about conceptual ideas of learners and appropriate instruction) (Kind and Chan, 2019). These knowledge domains are separable but correlated, as empirically demonstrated for several disciplines, including early literacy (König et al., 2022), mathematics education (Baumert et al., 2010), physics education (e.g., Sorge et al., 2019a), or biology education (e.g., Großschedl et al., 2019). Moreover, studies have shown that high levels of PCK among mathematics (Kunter et al., 2013), physics (Keller et al., 2017), and biology (Mahler et al., 2017) teachers have a beneficial effect on the quality of their classroom practice and thus on student learning. Hence, PCK is considered an essential resource for planning, implementing, and reflecting on instruction (Alonzo et al., 2019; Carlson and Daehler, 2019). PCK develops primarily over time as a result of learning and teaching experiences, as well as reflection on them (Van Driel and Berry, 2020). In this regard, however, the foundations should already be acquired during (predominantly theoretically oriented) university teacher training, which is also demanded by the standards for teacher education in several countries [e.g., Australia: New South Wales (NSW) Education Standards Authority, 2018; Canada: British Columbia Teachers’ Council (BCTC), 2019; Chile: Ministerio de Educación (MINEDUC), 2012; Germany: Sekretariat der Ständigen Konferenz der Kultusminister der Länder in der Bundesrepublik Deutschland (KMK), 2019]. Indeed, findings suggest a growth of pre-service teachers’ PCK during university studies (e.g., Kleickmann et al., 2013; Großschedl et al., 2019).

Because PCK is such an essential aspect of teachers’ professional competence, several attempts have been made to develop test instruments for measuring this construct as objectively, reliably, and validly as possible (e.g., Loughran et al., 2004; Park et al., 2018; Großschedl et al., 2019; She and Chan, 2022). Chan and Hume (2019) distinguish between two main approaches: (a) Paper-pencil tests (e.g., multiple-choice instruments) and (b) performance analyses, that is, measures which focus on teachers’ behavior in PCK-indicative situations (e.g., when planning a lesson). Performance analyses are both more time-consuming and associated with objectivity problems, but they are considered more action-valid (Chan and Hume, 2019). Concerning paper-pencil tests, open-ended instruments can capture a rather broad range of cognitions (Martinez, 1999), they often require a high level of interpretation in the scoring. Therefore, most test practitioners appreciate the advantages of multiple-choice instruments in terms of test economy and objectivity (Haladyna, 2004). However, developing multiple-choice tests is often challenging, for example, in terms of an accurate construct definition (i.e., an under- or overrepresentation of the construct must be avoided), the formulation of clear and unambiguous item stems and response options, or the development of a sufficient number of items to create a reliable instrument (Haladyna, 2004). Therefore, scholars have questioned the possibility to develop multiple-choice instruments to validly assess PCK (e.g., Smith and Banilower, 2015). This poses a challenge because closed-ended instruments are needed to investigate and establish quantitative relationships between PCK components among each other as well as between PCK and other variables.

Against this background, our study aims to investigate challenges that may arise when developing multiple-choice instruments to assess PCK in more detail. For this purpose, we exemplarily use a test instrument for assessing science teachers’ PCK about scientific reasoning (PCK_SR_) in order to explore differential response behavior within the framework of the expert-novice paradigm (De Groot, 1965). This paradigm assumes that experts more often correctly identify the quintessence of tasks due to a more elaborate knowledge network, whereas novices tend to focus on the tasks’ surface characteristics (Hogan et al., 2003). Accordingly, we consider the response behavior of researchers in biology education (experts) as well as that of pre-service biology teachers (novices). Based on theoretical assumptions, we analyzed the PCK_SR_ test scores, their variance, and the experts’ and novices’ explanations for choosing a response option in terms of surface and deep characteristics of the test items. This approach focusing on differential response behavior allows deriving specific recommendations for the construction of PCK-assessing multiple-choice instruments with better psychometric properties.

## 2. Theoretical background

The following sections describe the construct of PCK as well as its assessment in more detail, followed by a statement of how the expert-novice paradigm and the analysis of response behavior can lead to a better understanding of the functioning of test instruments.

### 2.1. Pedagogical content knowledge

**Figure 1:**
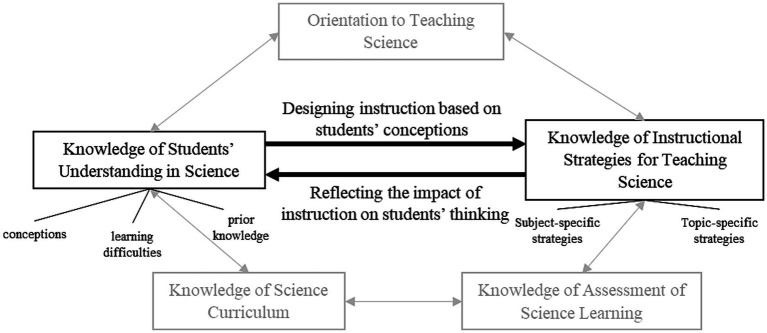
Pentagon-model of PCK; KSU and KISR as well as their connections are highlighted. Adapted from Park and Chen (2012) and Park and Oliver (2008).

As mentioned above, Shulman (1986, 1987) introduced PCK as an “amalgam” of PK and CK. Since this initial definition, several researchers have further developed the construct of PCK (Magnusson et al., 1999; Gess-Newsome, 2015; Carlson and Daehler, 2019; Neumann et al., 2019). A more recent definition specifies it as “the knowledge of, reasoning behind, and planning for teaching a particular topic in a particular way for a particular purpose to particular students for enhanced student outcomes” (Gess-Newsome, 2015, p. 36). Other scholars emphasize that PCK should be conceptualized as encompassing both (theoretical) knowledge and (practical) skills (Chan and Hume, 2019). In a broader sense, PCK thus covers several components that interact with each other. The “pentagon model of PCK” (Figure 1), for example, distinguishes five components of PCK: *Orientations to Teaching Science* (e.g., beliefs about the purpose of teaching science), *Knowledge of Instructional Strategies for Teaching* (e.g., subject-specific and topic-specific strategies), *Knowledge of Assessment of Science Learning* (e.g., about different kinds of performance evaluation), *Knowledge of Curriculum* (e.g., about its implications for teaching a specific subject), and *Knowledge of Students’ Understanding* (e.g., about [mis]conceptions and prior knowledge; Park and Oliver, 2008). Based on this model, a high level of PCK is characterized by close connections between these components (Park and Chen, 2012). Vice versa, less close or insufficient connections between components indicate poorly developed PCK (Park and Chen, 2012). The two components of *Knowledge of Students’ Understanding in Science* (KSU) and *Knowledge of Instructional Strategies for Teaching Science* (KISR), as well as their connection, are considered most crucial for effective teaching (Alonzo et al., 2012; Park and Chen, 2012; Jin et al., 2015). While the connection KISR→KSU reflects the impact of instructions on students’ conceptions, the connection KSU → KISR refers to addressing students’ (mis)conceptions when designing instruction.

Compared to Park and Oliver (2008), Carlson and Daehler (2019) argue that the construct of PCK does not merely consist of components, but of different realms of knowledge. In addition to collective PCK (cPCK), the canonical knowledge held by the community of researchers or practitioners in science education, they differentiate between personal PCK (pPCK), which describes the entire PCK an individual teacher possesses, and enacted PCK (ePCK), that is, the part of a teacher’s pPCK used during planning, teaching, and reflecting (Alonzo et al., 2019). In view of this, it is very important to always consider the specific realm in which teachers’ PCK is assessed when developing instruments (Sorge et al., 2019b; She and Chan, 2022). Consequently, assessing ePCK requires instruments that provide information about teachers’ PCK used during planning, teaching or reflecting (Alonzo et al., 2019). For instance, scholars suggest to analyze the quality of teachers’ written lesson plans for indicators of their PCK (Großmann and Krüger, 2023) or to use video-based instruments to assess science teachers’ PCK from their comments on authentic classroom teaching (She and Chan, 2022). In contrast, instruments that claim to measure pPCK require a cross-situational design. As a consequence, the different realms in which PCK is considered allow for different conclusions about the depth and the breadth of teachers’ PCK, respectively (Chan and Hume, 2019). In this manuscript, we will specifically address paper-pencil tests, which can be used to assess pPCK or cPCK (Carlson and Daehler, 2019).

### 2.2. Assessing PCK with paper-pencil tests

Several paper-pencil tests with different task formats exist to assess PCK. For example, some instruments consist of open-ended questions (e.g., CoRe by Loughran et al., 2004), self-report rating scales (e.g., an instrument by Tepner and Dollny, 2014), multiple-choice items (e.g., POSITT by Schuster et al., 2006), or mixed task formats (e.g., PCK-IBI by Großschedl et al., 2019). In instruments with open-ended questions, teachers are asked to answer in their own words. Such free-response tests aim more at drawing conclusions about the depth of teachers’ PCK (Smith and Banilower, 2015; Chan and Hume, 2019). In contrast, closed-ended multiple-choice questions require participants to select one correct response option from several given ones (Chan and Hume, 2019).

While open-ended questions instruments can capture a broader range of cognitions (Martinez, 1999), most test practitioners appreciate the advantages of multiple-choice instruments in terms of test economy and objectivity (Haladyna, 2004). Nevertheless, some researchers argue that PCK, due to its complexity, cannot be captured by multiple-choice items (e.g., Park and Oliver, 2008; Smith and Banilower, 2015). For this reason, some authors have developed instruments that comprise both closed- and open-ended questions to draw conclusions about both the breadth and the depth of PCK (Jüttner et al., 2013; Großschedl et al., 2019). However, as a consequence, most of these instruments require a quite long test duration (e.g., 24 items that take about 50 min to solve in Jüttner et al., 2013), and the data analysis requires equally long time due to the integration of open-ended items.

#### 2.2.1. Challenges in developing multiple-choice instruments

If tests consisting exclusively of multiple-choice items are used for reasons of test economy, a major challenge in the development of those instruments is to avoid a possible under- or overrepresentation of the construct. While the former is defined as “the degree to which a test fails to capture important aspects of the construct,” the latter is associated with construct-irrelevant variance, which “refers to the degree to which test scores are affected by processes that are extraneous to the test’s intended purpose”[American Educational Research Association (AERA) et al., 2014, p. 12]. Therefore, it is recommended to make sure that test items focus on narrowly defined aspects of PCK (Smith and Banilower, 2015). Such a clear focus and an adequate and unambiguous definition of PCK and its constituting aspects also help to avoid a possible underrepresentation of the target construct (Messick, 1996). At the same time, however, due to the definition of PCK as an “amalgam” of CK and PK (Shulman, 1987), there is also a risk of overrepresentation of the construct in terms of construct-irrelevant CK and/or PK variance, which means that “the assessment is too broad” (Messick, 1996, p. 5). In this regard, some multiple-choice instruments for assessing PCK have been criticized for including either tasks that require CK rather than PCK to solve them, or for focusing on teachers’ beliefs about teaching and learning by asking them about appropriate instructional interventions even though empirical evidence for the superiority of an instructional strategy over another strategy is often lacking (Smith and Banilower, 2015). For example, Schmitt (2016) used 19 items to assess chemistry teachers’ PCK about experimentation. Seventeen items describe a classroom situation and possible ways of (re)acting are to be rated on a five-point scale according to their appropriateness for the given situation. In contrast, the remaining two items of the instrument refer to scientific inquiry, asking teachers to sort the steps of scientific inquiry and to correctly assign specific statements to them. The final instrument has a reliability of *α* = 0.76, while validity was evaluated via expert ratings (Schmitt, 2016). Although the psychometric properties are acceptable, the two items referring to scientific inquiry rather capture CK than PCK, as they address declarative knowledge about the concept of inquiry and not knowledge about how to teach it (i.e., *scientific reasoning competencies*; Khan and Krell, 2019). Another example is Schuster et al.’s (2006) Pedagogy of Science Inquiry Teaching Test (POSITT) for the assessment of science teachers’ PCK about inquiry-based science teaching. The POSITT consists of multiple-choice items that relate to specific instructional settings. The response options for each item include different instruction-related actions (Schuster et al., 2006). In a second publication on this instrument, now called Pedagogy of Science Teaching Tests (POSTT), the authors changed its application: It is now to be used for formative assessment of science teachers’ orientations toward inquiry-based instruction (Cobern et al., 2014). This shift to a different construct during test development, from PCK to orientations, again highlights the challenge with items that were originally designed to capture PCK but are more likely to measure different constructs (Smith and Banilower, 2015).

To summarize, there are important benefits in multiple-choice items in terms of test economy and objectivity (Martinez, 1999; Haladyna, 2004). However, avoiding under- and overrepresentation of PCK is challenging (see Table 1). The result might be an instrument with only poor reliability and/or of questionable validity. Smith and Banilower (2015) therefore argued that the development of appropriate instruments for the assessment of teachers’ PCK will remain a challenge for science education. However, PCK is one of the core constructs in science teacher education, determining teaching quality and student learning outcome (Kunter et al., 2013; Keller et al., 2017; Mahler et al., 2017). Therefore, test instruments are highly needed to evaluate the success of teacher education programs (e.g., Karal and Alev, 2016) or teacher professional development (e.g., Sannert and Krell, 2023), for instance. Hence, exploring the challenges in developing multiple-choice instruments is likely to provide test developers with valuable insights and guidance for the successful development of multiple-choice instruments. We aim to contribute to this exploration with our study by analyzing differential response behavior in the framework of the expert-novice paradigm.

**Table 1:** Two major challenges in the development of multiple-choice PCK instruments.

Challenge	Description	Reference
Underrepresentation	The test does not cover all target aspects of PCK	e.g., Smith and Banilower (2015)
Overrepresentation	The test covers more than the target aspects of PCK (e.g., CK, beliefs)	e.g., Schuster et al. (2006)

### 2.3. Analyzing response processes within the framework of the expert-novice paradigm

Response processes refer to the cognitive, affective, and motivational mechanisms underlying responses to test items (Hubley and Zumbo, 2017). Thus, these mechanisms include both intentional, easily verbalizable processes and more unconscious, automatic reactions to test items. Think-aloud settings or interviews are particularly useful for capturing cognitive response processes. These methodological approaches analyze, for example, participants’ item interpretations, their response strategies, their applied knowledge, or the way they use item information (Ercikan and Pellegrino, 2017).

In general, two (overlapping) approaches to analyzing response processes can be distinguished: process-model interpretations and trait interpretations (Kane and Mislevy, 2017). Process-model interpretations are based on an exploration of the respondents’ task-solving process. In contrast, trait interpretations aim to investigate whether respondents apply those traits (e.g., knowledge, skills) claimed necessary to solve a task, or whether other traits are necessary (Kane and Mislevy, 2017). Both approaches can be used to evaluate the validity of test score interpretations (Ercikan and Pellegrino, 2017; Hubley and Zumbo, 2017).

In our study, we refer to the expert-novice paradigm to derive hypotheses about the test performance of two groups of respondents. Experts are defined as “individuals who exhibit reproducibly superior performance on representative, authentic tasks in their field” (Ericsson, 2006, p. 688). This definition implies that experts are expected to be better at solving tasks in their field than novices because of their superior knowledge and skills. Differences in how tasks and problems are dealt with can therefore be investigated on the basis of the expert-novice paradigm, which is commonly used in educational research. For example, Wolff et al. (2015) investigated differences in experts’ (experienced teachers) versus novices’ (pre-service teachers) representations of situations requiring classroom management. For this purpose, the descriptions of specific classroom situations were analyzed by using a coding scheme that included different kinds of representations, for example, thematic focus or cognitive processing. The results showed a significant difference in cognitive processing between experts and novices, with experts focusing on characteristics such as learning output and novices focusing rather on surface characteristics such as student behavior (Wolff et al., 2015). In another study by Krepf et al. (2018), the activated knowledge of experts (several criteria, e.g., at least 15 years of teaching experience) and novices (pre-service teachers) was analyzed during their evaluation of a videotaped lesson. While the experts activated their PCK, the novices (at best) activated limited CK and PK, which, moreover, were rarely related to each other. In addition, the experts could solve the problem better and describe their ways of doing so (Krepf et al., 2018). A related approach was chosen for the development of the PCK-IBI by Großschedl et al. (2019): The authors compared PCK test scores between experts (experienced teachers) and novices (pre-service teachers) to evaluate the criterion validity of their instrument. They found significantly higher levels of PCK for the experts as well.

However, there are not only quantitative differences between experts and novices, but also those related to the quality of knowledge and its use. For example, novices seem more likely to be distracted by surface characteristics of a problem or task, whereas experts more often understand the core idea, have a deeper understanding, and the ability to transfer this understanding to related problems (Hogan et al., 2003). In the specific context of PCK, experts and novices differ in terms of lesson planning, teaching, and reflection: While experts can adapt their instruction to the learning group’s knowledge comparatively easily, novices often use more rigid forms of interaction due to a lack of flexible, transferable knowledge (Hogan et al., 2003). Another study by Forsythe et al. (2022) reports on video analysis/reflection tasks in science teacher education. Here, the authors provided a framework for the analysis of surface and deep characteristics. They assumed that novices “might identify events that are superficial and disconnected from the lesson learning goals. […] Expert noticing focuses on events that are pivotal to achieving these goals” (p. 632).

Studies in other fields have also made the distinction between surface and deep characteristics of tasks. For the subject of chemistry, for example, Kozma and Russell (1997) showed that novices (undergraduate chemistry students) were often distracted by surface characteristics when solving chemical tasks, whereas experts (professional chemists) used their knowledge in terms of a deeper and more flexible understanding of chemical phenomena. Besides, surface and deep characteristics are important in educational research on the psychometric properties of items and test instruments (e.g., Opfer et al., 2012; Schnotz and Baadte, 2015; Krell, 2018). There is evidence suggesting that surface characteristics influence item difficulty in test-taking by novices, whereas experts can better recognize the deep characteristics of items and therefore respond more consistently to related ones (Clough and Driver 1986; Nehm and Ridgway, 2011). We take up these results to establish a distinction between *Surface Thinking* and *Deep Thinking*. *Surface Thinking* means that a respondent focuses on the surface characteristics of items when working on a test, whereas *Deep Thinking* means that the respondent recognizes the core idea of the task and relates to it in their response.

In summary, one can make several assumptions in light of the expert-novice paradigm about the response processes of experts and novices when solving multiple-choice items of a PCK test. These assumptions relate primarily to differences in the perception and cognitive processing of the items. For example, experts can be expected to achieve significantly higher test scores than novices because they have a higher level of PCK (Ericsson, 2006). In addition, novices can be expected to show more variance in their responses than experts, who are, vice versa, more likely able to answer items consistently by transferring their knowledge to related ones (Hogan et al., 2003; Wolff et al., 2015; Krepf et al., 2018). Finally, it can be assumed that experts tend to apply *Deep Thinking* more than novices, who, by contrast, are expected to apply more *Surface Thinking* when working on test items. This assumption is based on the consideration that experts have the PCK required to identify the core idea of an item (Hogan et al., 2003; Forsythe et al., 2022).

### 2.4. Aim of the study and hypotheses

To better understand and explain the challenges in developing multiple-choice tests to assess science teachers’ PCK, we exemplarily used an instrument to measure PCK_SR_ (Section 3.2.2) and considered the assumptions mentioned above to analyze differences in the response processes of experts and novices. This will provide valuable insights and guidance for the successful development of multiple-choice instruments. Specifically, we investigated the following research questions (RQ) and hypotheses (H):

*RQ1*: To what extent do experts achieve a higher proportion of correct responses in a PCK_SR_ test than novices?

*H1*: Experts reach a statistically significant higher proportion of correct responses in a PCK_SR_ test than novices.

*RQ2*: To what extent do experts’ answers in a PCK_SR_ test vary less than novices’ ones?

*H2*: Novices show significantly more variability in their answers in a PCK_SR_ test than experts.

*RQ3*: To what extent do experts and novices justify their choice of response options in a PCK_SR_ test differently?

*H3*: When experts justify their choice of response options in a PCK_SR_ test, their explanations are significantly more often indicative of *Deep Thinking*, while novices’ explanations are significantly more often indicative of *Surface Thinking*.

## 3. Materials and methods

The following sections give an overview of sample characteristics, the data collection, scoring, and the statistical methods applied to address our research questions and test our hypotheses. Our study was reviewed and approved by our local ethics committee (ID 2021_LI56) before any data collection took place.

### 3.1. Sample

A total of *N* = 20 participants took part in this study, who could be assumed to belong to one of two groups that differ significantly from each other on the construct of PCK_SR_. Accordingly, the two groups of participants in the study consisted of experts and novices. The experts were researchers in the field of biology education who were familiar with the construct of PCK_SR_, which means that they have obtained a PhD as well as conducted research and published in the field of PCK and scientific reasoning. The experts were individually invited to participate in this study via e-mail. The novices were undergraduate pre-service biology teachers from the authors’ university and were invited via e-mail lists of their university courses. Participation was voluntary.

Based on the considerations above (see Section 2.3), we expected undergraduate pre-service biology teachers to be PCK_SR_ novices because they just started their teacher university program and did not have the necessary learning opportunities to develop PCK since they are at the beginning of their university studies. At the same time, the experts in the field of biology education already did research and published about PCK and scientific reasoning, which makes it necessary to deal with it intensively.

A total of *n* = 10 experts agreed to participate in this study which is why we also randomly selected *n* = 10 novices to reach two groups of equal size. We considered this sample size as large enough to explore the challenges in developing multiple-choice PCK tests.

### 3.2. Data collection

A one-on-one interview was conducted and recorded with each participant via videoconference. The participants answered a total of 12 multiple-choice items from a test designed to assess PCK_SR_ during the interviews.

#### 3.2.1. Interview protocol

At the beginning of the interview, the participants were informed about the purpose of the study, data collection, and data management. After consenting to take part in the study, it was explained that the task will be to answer 12 multiple-choice items from a test designed to assess PCK_SR_ and to verbally explain the responses.

After answering an item, the participants justified why they chose that particular response option and explained why they did not choose another option, respectively. If a participant could not decide on a response option, they were asked to explain why no option seemed right. Subsequently, they did not have to select a response option.

This process was repeated analogously for all items. After all items were answered, the interview ended.

#### 3.2.2. Multiple-choice test

All items of the multiple-choice test comprised one attractor (correct response option) and two distractors (incorrect response options), as suggested by Rodriguez (2005). After completing the test, the participants were asked to justify their choice of response options and to explain why they did not choose another option, respectively.

As the multiple-choice instrument was designed to assess PCK_SR_, it refers to teaching scientific reasoning, an essential part of science and biology education (Mathesius et al., 2016; Khan and Krell, 2019). Biology teachers are expected to foster their students’ scientific reasoning competencies to enable them to participate in discussions and decision-making on current social challenges (Osborne, 2013). Therefore, scientific reasoning competencies are emphasized in science educational standards and curricula in various countries worldwide [e.g., Australia: Australian Curriculum, Assessment and Reporting Authority (ACARA), 2022; Canada: Ministry of Education, 2008a,b; Chile: Ministerio de Educación (MINEDUC), 2015, 2019; Germany: KMK, 2005, 2020]. An essential prerequisite for promoting such competencies among students is that their teachers have an appropriate level of PCK_SR_ which relates to methodological and pedagogical competencies for teaching scientific reasoning (Justi and van Driel, 2006; Günther et al., 2019).

Based on Smith and Banilower’s (2015) recommendation to focus on capturing narrowly defined PCK aspects, the items of our PCK_SR_ test refer to only two components of PCK (KSU, KISR) as well as their connections (KISR→KSU, KSU → KISR). Empirically, KSU and KISR have been shown the most crucial components for effective science teaching (Alonzo et al., 2012; Park and Chen, 2012; Jin et al., 2015), which is why existing test instruments mainly focus on one (e.g., Sadler et al., 2013) or both of them (e.g., Park et al., 2018; Großschedl et al., 2019). As scientific reasoning involves numerous different activities, there is not yet a standard definition of the construct (Krell et al., 2022). Rönnebeck et al. (2016) found, among others, three highly relevant scientific reasoning skills: (1) planning and conducting investigations, (2) generating hypotheses, and (3) using scientific models. These skills also form the dimensions of our PCK_SR_ instrument (Table 2).

**Table 2:** Systematic overview of the 12 multiple-choice items, developed to assess three skills of scientific reasoning, two PCK components, and their connections, respectively.

	Planning and conducting investigations	Generating hypotheses	Using scientific models
KISR	item #1	item #2	item #3
KSU	item #4	item #5	item #6
KISR→KSU	item #7	item #8	item #9
KSU → KISR	item #10	item #11	item #12

**Figure 2:**
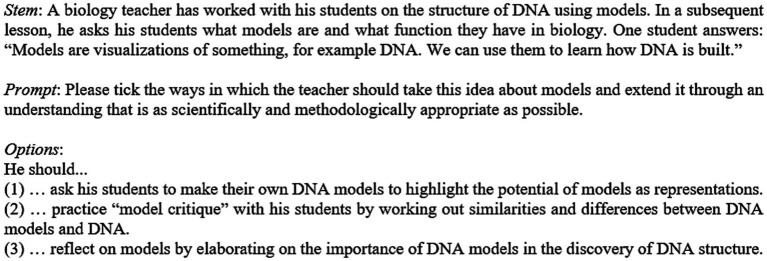
Sample item (Item 12). In response options 1 and 2, models are portrayed as mere visualizations according to the classical conception of students (Upmeierzu Belzen et al., 2019). For this reason, the first two response options do not describe appropriate ways to guide students’ ideas toward an adequate scientific understanding. In contrast, the third answer option describes an instructional intervention that emphasizes the role of models as epistemic tools in the context of scientific reasoning (Knuuttila, 2011; Göhner and Krell, 2022).

After clarifying the construct to be assessed by choosing the two PCK components (Park and Oliver, 2008) and three crucial scientific reasoning skills (Rönnebeck et al., 2016), the items were developed in an elaborated sequence: First, the general design of the items was conceptualized. Each item of our PCK_SR_ instrument consists of a stem, the prompt, and three response options (Figure 2). The prompts are identical for all related items (e.g., the KISR items 1, 2, and 3) to reduce the possible influence of construct-irrelevant sources of variance (Messick, 1996). Moreover, all stems are nearly of equal length and contain identical pieces of information, for example, an outline of students’ skills, the topic, or the intended learning outcome (for an example, see item 12 in Figure 2). After the item stems had been determined, they were first presented in the form of open-ended questions to a sample of *n* = 14 pre-service biology teachers in order to generate response options that were as ecologically valid as possible and that corresponded to the knowledge and skills of the target group (Haladyna, 2004). Afterward, we developed both correct (attractors) and wrong response options (distractors). They are all about the same length and of similar phrasing. Such standardization is intended to increase the likelihood that respondents will think about the semantic content of a response option, rather than being attracted by superficial cues. Subsequently, the items were administered to two samples of pre-service biology teachers to collect empirical validity evidence [e.g., evidence based on internal structure; American Educational Research Association (AERA) et al., 2014]. The item development and the result of the validation study are reported in Lieberei et al. (2023), which is why we do not report this in more detail within the current article. Figure 2 shows a sample item that relates to the connection KSU → KISR on the “using scientific models” dimension. The full instrument is available upon request from the last author.

### 3.3. Scoring

To address RQ1 and RQ2, we analyzed whether participants selected the attractor or one of the two distractors. If a participant decided not to answer an item (e.g., because they considered the response options ambiguous or were unsure about the correct answer but did not want to fail), this was treated as a missing response that was excluded from subsequent data analyses. To address RQ3, all interviews were first transcribed word-by-word, as described in Dresing and Pehl (2018). In the second step, we assigned the participants’ justifications for choosing or not choosing response options for each item. If a justification referred to more than one response option at once, it was assigned to all affected ones. If a justification could not be assigned to any response option, it was assigned to the item in general. In a third step, we further analyzed the reorganized transcripts by means of a coding scheme in a structured qualitative content analysis (Schreier, 2014). Its development was carried out deductively-inductively (Schreier, 2012). In this process, we first deductively developed an initial version in line with the distinction between surface and deep characteristics (Hogan et al., 2003; Krepf et al., 2018) which exclusively included the main categories of *Surface Thinking* and *Deep Thinking*. This preliminary coding scheme was then tested step by step during the interviews with the participants to check whether it reflected their answers in a sufficiently exhaustive way. If an answer could no longer be easily classified into the existing categories (e.g., because something new was addressed), the coding scheme was expanded by creating a new (sub)category (Schreier, 2012). Subsequently, 10% of the interviews (one novice and one expert interview) were coded by both the first author of this article and a trained student assistant. Discrepancies between the two coders were discussed afterward to refine the category system.

The final coding scheme comprised the two main categories *Surface Thinking* and *Deep Thinking* as well as 16 subcategories (Table 3). Some of the ten inductively developed subcategories of *Surface Thinking* have also been used in previous studies (e.g., *CK* or *PK*; Krepf et al., 2018), while others are theory-based (e.g., *Item Stem*, based on Kunter and Trautwein, 2013). The six subcategories of *Deep Thinking* were deductively derived from the pentagon model (Section 2.1; Park and Oliver, 2008; Park and Chen, 2012). The complete version of the coding scheme can be found in the Supplementary material of this article.

**Table 3:** Short version of the coding system (the complete version can be found in the Supplementary Table S1).

Main category	Sub category	Intercoder agreement (*κ*)
Surface Thinking	Item Stem – Student Characteristics	0.82
	Item Stem – Topic	0.48
	Item Stem – Teaching Methods	0.54
	Personal Experience	1.00
	Intuition	0.79
	Wrong Interpretation	0.70
	Misconception	−0.02
	Pedagogical Knowledge (PK)	0.76
	Content Knowledge (CK)	0.79
	No Relation to Scientific Reasoning	0.62
Deep Thinking	Orientations to Teaching Science	not coded
	Knowledge of Students’ Understanding	0.83
	Knowledge of Science Curriculum	1
	Knowledge of Instructional Strategies and Representations of Teaching Science: Topic-Specific-Strategies	0.00
	Knowledge of Instructional Strategies and Representations of Teaching Science: Subject-Specific-Strategies	0.79
	Knowledge of Assessment of Science Learnings	not coded

Categories that did not occur in the selection of transcripts for the intercoder agreement are marked as “not coded.”

As part of the structured qualitative content analysis, the participants’ responses were assigned to the 16 subcategories of the coding scheme. Here, the shortest coding units were less than one sentence, while the longest were less than one paragraph. If a participant’s justification referred to more than one response option, it was coded in the same category for all affected response options. If a participant had changed their choice of a response option, only the justification for their final choice was coded. Statements that were no justifications for choosing a response option (e.g., an analysis of the item context) were not coded. We then built sum scores for each participant and each item (i.e., 1 = subcategory was coded, 0 = subcategory was not coded). Therefore, a maximum sum score of 12 could be achieved on each subcategory.

Inter-coder agreement was ensured by following recommendations of Wirtz and Caspar (2002). To do so, 20% of the transcripts were coded independently by a second rater (the same trained student assistant as before). To increase the probability of representativeness, *n* = 2 interviews of novices and experts each were randomly selected (O’Connor and Joffe, 2020). The intercoder agreement was then calculated separately for each subcategory (Table 3). For most subcategories, Cohen’s Kappa indicated a substantial (0.61 ≤ *κ* ≤ 0.80) to almost perfect (*κ* ≥ 0.81) intercoder agreement (Landis and Koch, 1977). For the two subcategories *Item Stem – Topic* and *Item Stem – Teaching Methods*, a moderate agreement was reached (0.41 ≤ *κ* ≤ 0.60), while in two cases a slight (0.00 ≤ *κ* ≤ 0.20) or poor agreement (*κ* < 0.00) was found (*Misconception* and *Knowledge of Instructional Strategies and Representations of Teaching Science: Topic-Specific-Strategies*); the latter likely as a result of only a few codings in the respective subcategories (see Table 4 for the number of codings in the total sample). On average, an intercoder agreement of *κ* = 0.72 resulted for the subcategories of *Surface Thinking* while one of *κ* = 0.87 resulted for the subcategories of *Deep Thinking*. All discrepancies between the two coders could be resolved by discussion.

**Table 4:** Overview of the codings.

Category	Number of codings		
	Experts	Novices	Total sample
Surface Thinking			
Item Stem – Student Characteristics	19	24	43
Item Stem – Topic	2	4	6
Item Stem – Teaching Methods	22	36	58
Personal Experience	1	11	12
Intuition	22	66	88
Wrong Interpretation	44	68	112
Misconception	1	4	5
Pedagogical Knowledge (PK)	8	25	33
Content Knowledge (CK)	10	6	16
No Relation to Scientific Reasoning	22	40	62
**Sum**	151	284	435
Deep Thinking			
Orientations to Teaching Science	12	0	12
Knowledge of Students’ Understanding	70	53	123
Knowledge of Science Curriculum	2	0	2
Knowledge of Instructional Strategies and Representations of Teaching Science: Topic-Specific-Strategies	2	1	3
Knowledge of Instructional Strategies and Representations of Teaching Science: Subject-Specific-Strategies	77	43	120
Knowledge of Assessment of Science Learnings	0	0	0
**Sum**	163	97	260

### 3.4. Statistical analyses

To address RQ1 and RQ2, we first calculated the proportion of correct responses by dividing the number of correct answers (max. 12) by the total number of items answered (max. 12). This procedure was chosen to account for the fact that some participants had not answered some questions (possibly for reasons unknown to us; see Section 3.3). We then tested the proportion of correct answers calculated in this way for normal distribution in both groups before running a one-tailed *t*-test for independent groups (RQ1) as well as Levene’s test for equality of variances (RQ2).

To address RQ3, the sum scores in each subcategory of *Surface Thinking* and *Deep Thinking* were again summed up to build a total score for these two main categories. These total scores were normally distributed in both groups in the case of *Deep Thinking*, but not in that of *Surface Thinking* (Shapiro–Wilk test). For this reason, a Mann–Whitney U test was conducted to address RQ3.

The interview coding and qualitative analyses were done with MAXQDA 2022; the quantitative analyses with IBM SPSS Statistics 26. Effect sizes were calculated using an online tool by Lenhard and Lenhard (2016).

## 4. Results

In the following, the results of the statistical analyses are presented separately for each of the research questions, additionally illustrated in each case by corresponding text examples from the interview transcripts. Table 5 provides an overview of the descriptive statistics for each item of the PCK_SR_ test. Item 12 turned out to be the item with the largest difference between experts and novices in terms of the proportion of correct answers, while item 7 involved the largest difference in the variability of answers (in terms of standard deviation).

**Table 5:** Descriptive statistics for each item of the PCK_SR_ items and the full test score (*M* = mean score, *SD* = standard deviation).

		Items												Test score
		#1	#2	#3	#4	#5	#6	#7	#8	#9	#10	#11	#12	
Experts	*M*	0.70	0.44	0.38	0.80	0.90	0.89	0.89	0.80	0.70	0.33	0.11	0.89	0.67
	*SD*	0.46	0.50	0.48	0.40	0.30	0.31	0.31	0.40	0.46	0.47	0.31	0.31	0.12
Novices	*M*	0.50	0.60	0.20	0.60	0.70	0.80	0.60	0.50	0.70	0.40	0.30	0.30	0.52
	*SD*	0.50	0.49	0.40	0.49	0.46	0.40	0.49	0.50	0.46	0.49	0.46	0.46	0.20
Total sample	*M*	0.60	0.53	0.28	0.70	0.80	0.84	0.74	0.65	0.70	0.37	0.21	0.58	0.59
	*SD*	0.49	0.50	0.45	0.46	0.40	0.36	0.44	0.48	0.46	0.48	0.41	0.49	0.18

### 4.1. RQ1: To what extent do experts achieve a higher proportion of correct responses in a PCK_SR_ test than novices?

The *t*-test revealed that the experts reached a statistically significant higher proportion of correct answers (*M* = 0.67, *SD* = 0.12) than the novices (*M* = 0.52, *SD* = 0.20), *t*(18) = 2.03, *p* = 0.029. This corresponds to a large effect, *d* = 0.91 (Cohen, 1988).

The following parts of the interview transcripts, which refer to item 12 (Figure 2), can be used to consider potential reasons for the group differences found. Most of the experts (8 out of 9 [1 missing answer]) identified the attractor correctly and were able to justify their choices accordingly, while most of the novices (7 out of 10) ruled out the attractor as the correct answer.

Two examples of experts’ justifications:

*I would say the third one is [the right answer]. […] In my opinion, this item addresses that this student did not understand that the model is an element in the process of scientific inquiry. [The third response option] […] specifically addresses this. Therefore I think this answer is most convincing.* – (Expert 4) [Coding: *“Knowledge of Students’ Understanding” and “Knowledge of Instructional Strategies and Representations of Teaching Science: Subject-Specific-Strategies”*]

*Here, students have an […] idea of models that is not at all predominant in biology. The point is that the students learn that models are not only there for illustration, but are also used to gain knowledge and I see this in answer option (3) […]. There, the gain in knowledge becomes more apparent and the students realize […] [that] the model was previously used to elucidate the structure in the first place.* – (Expert 9) [Coding: *“Knowledge of Students’ Understanding” and “Knowledge of Instructional Strategies and Representations of Teaching Science: Subject-Specific-Strategies”*]

Two examples of novices’ justifications:

*‘Reflect on models’- I don’t find that important. So I would rather take (2) [because] I really don’t know what [the way in] (3) is supposed to do. […] I don’t know, I find that somehow strange. And then reflecting on models- I don’t find that important either. So I would rather take (2). […] And with (3)- […]. Yes, I don’t really know what the point is. […] Yes, I don’t know, this is somehow strange.* – (Novice 4) [Coding: *“No Relation to Scientific Reasoning” and “Intuition”*]

*I would definitely not choose the third one, because I think it only promotes the misconception. [This is because] it says ‘reflect on models by elaborating on the importance of DNA models in the discovery of DNA structure’ which is exactly what they already know. […] This would confirm the student in her idea that models are [visualizations of something and] used to see the structure [of DNA], because by doing [the way in answer option (3)] in the lesson, only the structure of the models is addressed. So I think you have to go into it further.* – (Novice 1) [Coding: *Wrong Interpretation*]

### 4.2. RQ2: To what extent do experts’ answers in a PCK_SR_ test vary less than novices’ ones?

**Figure 3:**
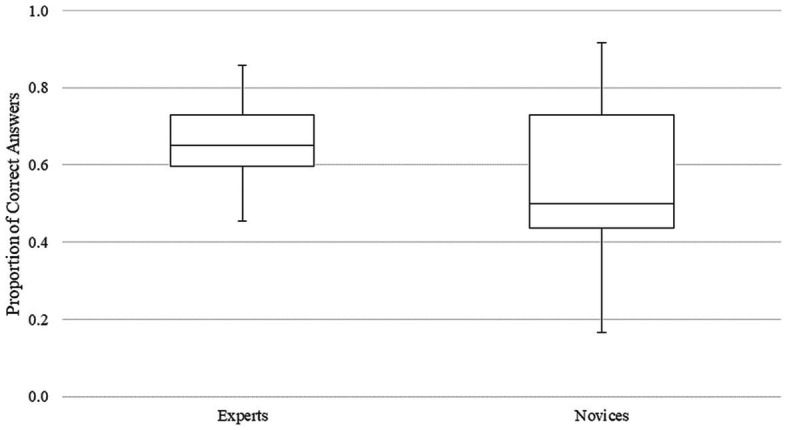
Boxplots of the proportions of correct answers.

Levene’s test revealed that the variability in the experts’ responses (s^2^ = 0.04) was not significantly different from the variability in the novices’ responses (s^2^ = 0.02), *F*(1, 18) = 0.94, *p* = 0.340. However, a clear trend can be seen in nominal terms (Figure 3).

**Figure 4:**
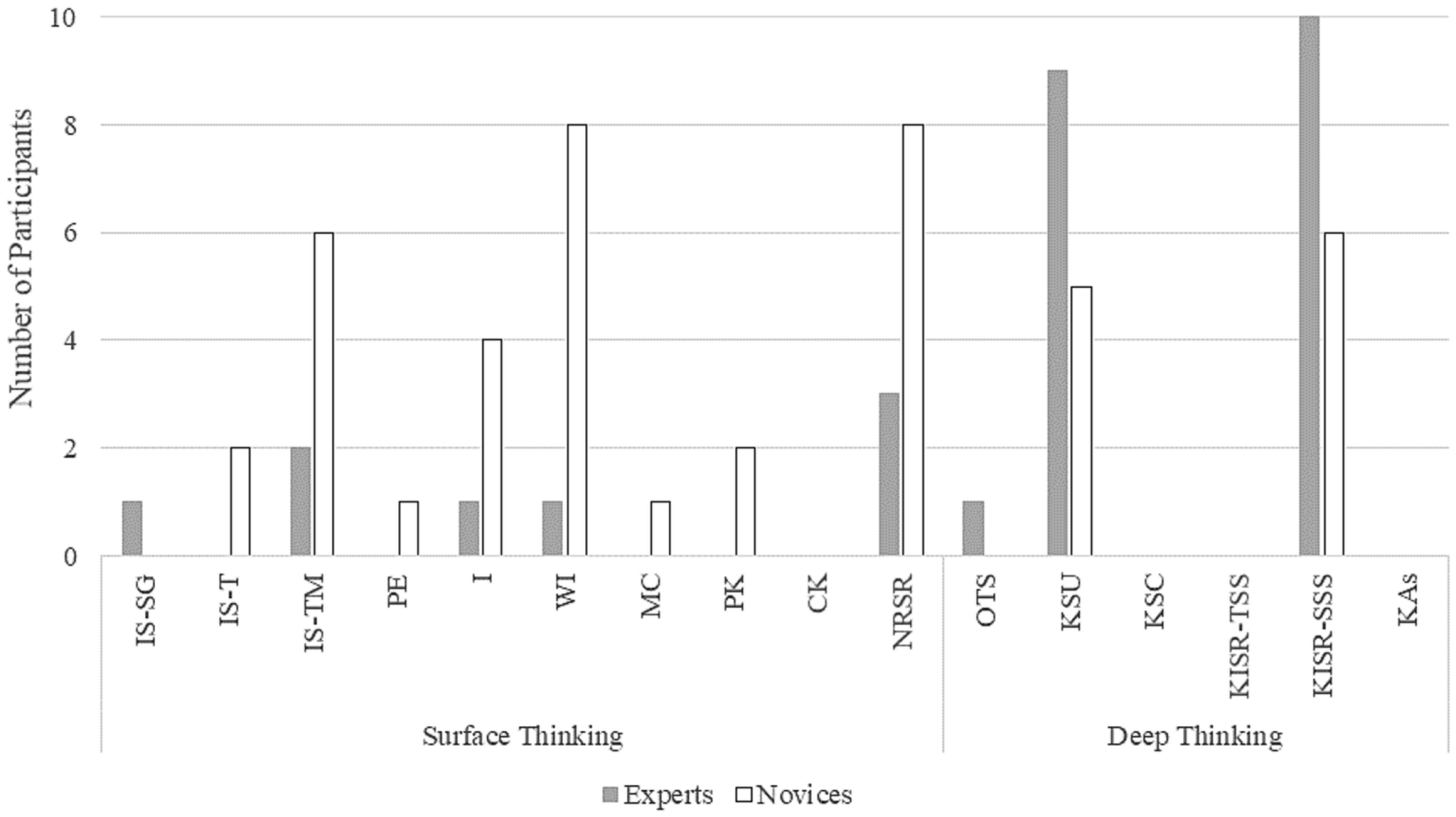
Comparison of the number of experts and novices whose justifications for choosing a response option was assigned to a specific subcategory of *Deep Thinking* and *Surface Thinking*. IS-SG, Item Stem – Study Group; IS-T, Item Stem – Topic; IS-TM, Item Stem – Teaching Methods; PE, Personal Experience; I, Intuition; WI, Wrong Interpretation; MC, Misconception; PK, Pedagogical Knowledge; CK, Content Knowledge; NRSR, No Relation to Scientific Reasoning; OTS, Orientations to Teaching Science; KSU, Knowledge of Students’ Understanding; KSC, Knowledge of Science Curriculum; KISR-TSS, Knowledge of Instructional Strategies and Representations of Teaching Science: Topic-Specific-Strategies; KISR-SSS, Knowledge of Instructional Strategies and Representations of Teaching Science: Subject-Specific-Strategies; KAS, Knowledge of Assessment of Science Learnings.

This result corresponds to the pattern of justifications for choosing a response option. Whereas the experts’ justifications could be mainly assigned to only two subcategories of *Deep Thinking* (*Knowledge of Students’ Understanding* and *Knowledge of Instructional Strategies and Representations of Teaching Science: Subject-Specific-Strategies*), the novices’ justifications point to numerous different considerations in their choices of response options (Figure 4). These differences probably reflect the higher variability in the proportion of correct answers in the novices group.

The following parts of the interview transcripts, which again refer to item 12 (Figure 2), may indicate potential reasons for the group differences found.

Two examples of experts’ justifications:

*Answer option (2) would be too simple in my opinion. The students would only learn what they already know, which is that [models] are visualizations of something which we can use to learn how DNA is built. In answer option (2), they would only discuss, for example, aspects like redundancy and size, but that would have no additional knowledge gain, because they could figure this out on their own.* – (Expert 1) [Coding: *“Knowledge of Students’ Understanding” and “Knowledge of Instructional Strategies and Representations of Teaching Science: Subject-Specific-Strategies”*]

*The point in answer option (3) is that models should not only be seen as a teaching method, as a visualization, but that it should really help for scientific inquiry, […] which means that the students should learn the purpose for which models are used in science […].* – (Expert 5) [Coding: *“Knowledge of Students’ Understanding” and “Knowledge of Instructional Strategies and Representations of Teaching Science: Subject-Specific-Strategies”*]

Four examples of novices’ justifications:

*[The way described in answer option (1) would not be the right way.] When the student sees the visualization, her own DNA model would be very similar to the model that she already knows and she would still have the same problem with understanding [the function of models].* – (Novice 9) [Coding: *“Knowledge of Students’ Understanding” and “Knowledge of Instructional Strategies and Representations of Teaching Science: Subject-Specific-Strategies”*]

*Okay. (…) I would take (2) in any case. […] What she says is correct, you can see how the DNA is built, depending on how simplified it is. And then I would just go a step further and ask [the student] […] what’s missing in the model, [so that she can practice ‘model critique’].* – (Novice 4) [Coding: *Item Stem – Teaching Methods*]

*Answer option (3), […] that is simply reflecting on the learning process and seeing how important the model was for the understanding of the structure. But I think in the end you get the same result as with answer option (2), but there the learning process […] is not as given as with answer option (3) because […] you give them the information there and they work it out for themselves in answer option (2). I would exclude answer option (3) because the students are already given the knowledge acquisition and they cannot make it for themselves in their own thought process.* – (Novice 8) [Coding: *“Wrong Interpretation” and “Item Stem - Teaching Methods”*]

*I also did not choose answer option (3), […] even though it is an important and correct way. In order to take the student’s idea, I think that’s missing the point a bit, because it’s already about the discovery of the DNA structure, where I don’t know how much has already been said about the research of DNA with the students. […] Actually, you teach the students what the current knowledge is and […] how you got to that. They should not throw [the knowledge] all together and then go into an exam with the knowledge from the 18^th^ century. They should really know what is assumed nowadays. That’s why I think that practicing ‘model critique’ [in answer option (2)] is much more beneficial to extent the students’ idea through an understanding that is as scientifically and methodologically appropriate as possible than answer option (1) or (3).* – (Novice 3) [Coding: *No Relation to Scientific Reasoning*]

### 4.3. RQ3: To what extent do experts and novices justify their choice of response options in a PCK_SR_ test differently?

A total of 695 codings were done in the analysis of the *N* = 20 interviews (*n* = 435 codings of *Surface Thinking*, *n* = 260 codings of *Deep Thinking*). A detailed overview of the codings can be found in Table 4. The most frequently coded subcategory of *Surface Thinking* was *Wrong Interpretation* for both, experts and novices. The most frequently coded subcategory for *Deep Thinking* was KSU, also for both, experts and novices.

**Figure 5:**
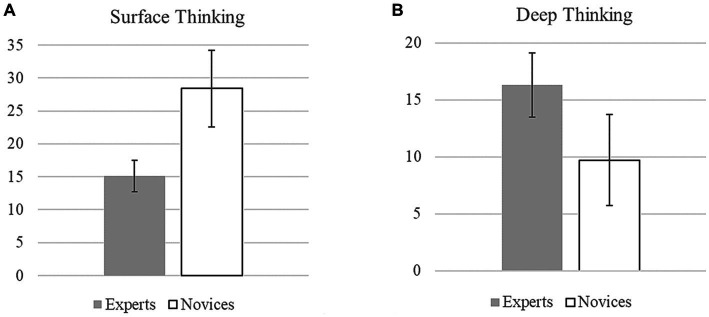
Sum of the codings (± 1 standard deviation) of **(A)** *Surface Thinking* and **(B)** *Deep Thinking* for novices and experts compared. The total sum scores for **(A)** *Surface Thinking* are generally higher than those for **(B)** *Deep Thinking* because of the different numbers of subcategories (10 versus only 6; see Table 4).

The total sum scores were used to compare experts and novices with regard to the categories of *Surface Thinking* and *Deep Thinking*. The Mann–Whitney U test revealed a significantly higher level of *Deep Thinking* for experts (*Sum* = 163) than for novices (*Sum* = 97), *U* = 5.50, *z* = 3.39, *p* < 0.001, *d* = 2.28, whereas novices showed significantly more often *Surface Thinking* (*Sum* = 284) than experts (*Sum* = 151), *U* = 0.00, *z* = 3.79, *p* < 0.001, *d* = 3.16 (Figure 5).

The different levels of experts’ and novices’ *Surface Thinking* and *Deep Thinking* can also be illustrated in the participants’ justifications for choosing a response option of item 12. Two experts, for example, said:

*However, I would now tend towards answer option three, […] because this addresses exactly the point which the student does not perceive. She states that models are pure visualizations of reality, but does not consider that insights can also be gained through such model considerations.* – (Expert 8) [Coding: *“Knowledge of Students’ Understanding” and “Knowledge of Instructional Strategies and Representations of Teaching Science: Subject-Specific-Strategies”*]

*Making DNA models to highlight the potential [in answer option (1)] rather promotes the perspective [of models as] representations which would reinforce the idea [of the student]. Practicing ‘model critique’ [in answer option (2)] is also a medial perspective and would not lead further in terms of scientific methodology. I’ll take answer option (3) then. There you have a historical example, Watson and Crick, where you can draw the importance of DNA models [in the discovery of DNA structure] over time, [by showing] the changes with more increase in knowledge, also from other researchers.* – (Expert 6) [Coding: *“Knowledge of Students’ Understanding” and “Knowledge of Instructional Strategies and Representations of Teaching Science: Subject-Specific-Strategies”*]

Here the experts argue with both, *Knowledge of Students’ Understanding* and *Knowledge of Instructional Strategies and Representations of Teaching Science: Subject-Specific-Strategies*. This corresponds exactly to the theoretical background of the item, which was intended to address the interaction KSU → KISR (Table 2). One expert also addressed *Orientations to Teachings Science* in their justification:

*That’s easy, [I don’t choose] answer option (1). Models as representations, this would promote the idea of the student, because the visualization character of models is promoted by this, which is level 1 in the competence model [by Upmeierzu Belzen et al. (2019)]. [For answer option (2)] […], this is similar to what I said earlier. It is not about practicing ‘model critique’ within the meaning of an understanding that is scientifically and methodologically as appropriate as possible, because that is also not on level 3 of the competence model [by Upmeierzu Belzen et al. (2019)]. But [the lesson] should be about reflecting on the purpose of models. In this case, what purpose the model has in order to discover the phenomenon of DNA structure.* – (Expert 3) [Coding: *“Knowledge of Students’ Understanding” and “Knowledge of Instructional Strategies and Representations of Teaching Science: Subject-Specific-Strategies”, and “Orientation to Teaching Science”*]

Regarding *Surface Thinking*, a novice, for example, justified their answer as follows:

*But I think that practicing ‘model critique’ is just a bit more plausible and perhaps sticks a bit more [than the way in answer option (3)]. I don’t know why at the moment.* – (Novice 7) [Coding: *Intuition*]

This is a representative example of the subcategory *Intuition* because it is not a justification based on the PCK_SR_ of the participant. Instead, the novice cannot justify why they chose this answer option. Many novices, on the other hand, did not interpreted the item in the intended way. For example:

*I don’t think they have to reflect on models and work out the importance of DNA models in the discovery of DNA structures [as in answer option (3)]. That is already the idea that is in the students’ heads, that you can learn with it how the DNA is built and I think that’s also a very intuitive idea. So I think they don’t have to reflect on it.* – (Novice 1) [Coding: *Wrong Interpretation*]

Other novices justify without reference to scientific reasoning. For example, the following participant only argues that model criticism is the right way. They does not argue with scientific reasoning competencies.

*I would choose answer option (2), simply because the student currently has the idea that models represent the truth and how the DNA is structured. But just because of the size that can’t be right. And I think it’s important to make it clear to the students that [a model] is not a biologically accurate visualization of something […] [and] give them therefore a critical view of it […].* – (Novice 3) [Coding: *No Relation to Scientific Reasoning*]

## 5. Discussion

With our study, we aimed to contribute to a better understanding of the challenges in developing multiple-choice instruments to assess science teachers’ PCK. For this purpose, we exemplarily used an instrument for the assessment of PCK_SR_ and considered assumptions from the expert-novice paradigm to analyze differences in the response processes of experts and novices. Our research questions referred to potential group differences regarding the proportion of correct responses (RQ1), the variability in that proportion (RQ2), and the frequency to which justifications for choosing a response option indicate either *Surface Thinking* or *Deep Thinking* (RQ3). While the corresponding hypotheses 1 and 3 could be empirically supported by the results, the difference postulated in hypothesis 2 was only found at a nominal, but not at a statistically significant level.

### 5.1. RQ1: To what extent do experts achieve a higher proportion of correct responses in a PCK_SR_ test than novices?

The significantly higher proportion of correct responses among the experts is in line with previous studies which showed that the knowledge of novices is on a significantly lower level (Hogan et al., 2003; Krepf et al., 2018; Großschedl et al., 2019). This assumption is also supported by the analysis of the qualitative data. For item construction in instruments to measure PCK, this means that the target group must be given appropriate consideration. Methodically, the proportion of correct answers corresponds to the mean item difficulty (Schweizer and DiStefano, 2016). According to test-theoretical recommendations, the individual items’ difficulties should range between 0.20 (20% of participants solve the item correctly) and 0.80 (80% of participants solve the item correctly), whereas, across all items, a medium item difficulty is to be aimed for (Schweizer and DiStefano, 2016). Given that, our PCK_SR_ instrument seems perfectly suited to capture the construct in undergraduate pre-service teachers (Table 5), but it seems less suited to assess experts’ PCK_SR_ because their average probability of correctly answering an item is 0.67, which is clearly above the recommendation of a medium item difficulty. In this context, however, an immediate question arises about the proportion of correct answers likely to be achieved by individuals with a PCK_SR_ level ranging between that of novices and experts (e.g., master’s students or teachers who have just completed their teacher education program). Even though PCK develops primarily over time as a result of practical experiences and reflection on them (van Driel and Berry, 2020), some studies have shown an increase in PCK among students during the university studies (e.g., Kleickmann et al., 2013). It is therefore to be expected that novices will also achieve a higher test score as they progress in their university studies. For the development of multiple-choice PCK instruments for pre-service science teachers, it also makes a difference what level of education they have.

### 5.2. RQ2: To what extent do experts’ answers in a PCK_SR_ test vary less than novices’ ones?

The (statistically insignificant) larger variability in the novices’ answers is in line with previous studies which have already shown experts more likely capable to answer items consistently by transferring their knowledge to related ones (Hogan et al., 2003; Wolff et al., 2015; Krepf et al., 2018). The analysis of the qualitative data also supports this assumption. This is an expected result since novices usually do not have sufficient PCK to answer the items consistently. The novices in this study were undergraduate pre-service biology teachers and, consequently, had only few previous learning opportunities related to PCK as part of their university studies. It is to be expected that they will also become more consistent in their response behavior as their level of education increases. This could also be shown in previous studies (Kleickmann et al., 2013).

This finding is important for instrument development, because a higher variance results in a higher number of items needed to reach appropriate discrimination and internal consistency (Kline, 2015). Hence, instruments to assess experts’ PCK would require fewer items than those administered among novices.

### 5.3. RQ3: To what extent do experts and novices justify their choice of response options in a PCK_SR_ test differently?

The significantly higher levels of experts’ *Deep Thinking* and novices’ *Surface Thinking* are in line with previous studies showing that surface characteristics influence item difficulty in test-taking by novices, whereas experts can better recognize the deep characteristics of an item (Clough and Driver, 1986; Nehm and Ridgway, 2011). From an assessment perspective, the influence of item surface characteristics on item difficulty is a construct-irrelevant source of variance (Messick, 1996), which, in turn, threatens the validity of test score interpretation. Hence, the comparatively high level of *Surface Thinking* among novices may indicate limited validity of the PCK_SR_ test scores in this group, whereas experts’ test scores may be validly interpreted as indicators for their PCK_SR_. These findings highlight the need to conduct sample-specific validation studies for PCK instruments and to not conceptualize validity as a fixed, ubiquitous characteristic of a test instrument [American Educational Research Association (AERA) et al., 2014].

In summary, the findings thus consistently support the validity of our PCK_SR_ instrument. Nevertheless, we will continue to optimize it based on revision suggestions from the experts. We expect that this will further improve the psychometric quality.

### 5.4. Conclusion

In conclusion, this study aimed to understand and explain challenges regarding an under- or overrepresentation of PCK in the development of multiple-choice PCK instruments, which poses a challenge to the science education research community as closed-ended instruments are required to quantify relationships among PCK components as well as with other variables. Based on the expert-novice paradigm, it was investigated to what extent experts provide a higher proportion of correct responses and show less variability in their answers than novices, as well as to what extent experts and novices justify their choice of response options to an exemplarily used PCK_SR_ multiple-choice instrument. It could be statistically shown that experts reach a significantly higher proportion of correct answers than novices. Furthermore, it could be shown that experts’ justifications were significantly more often indicative of *Deep Thinking*, while novices’ justifications were significantly more often indicative of *Surface Thinking*. However, a difference in the answers’ variability between experts and novices was only found at a nominal, but not at a statistically significant level. Keeping these conclusions in mind can support the development and interpretation of PCK instruments. The results of our study show that the analysis of response processes using the expert-novice paradigm can help to understand certain challenges in the context of test construction and to optimize this process accordingly.

### 5.5. Implications

Finally, the following implications can be given: First, care should be taken to ensure that instruments address only a few, specific PCK aspects (Smith and Banilower, 2015). Second, our results suggest it may be helpful to also consider the target group of a test, even during piloting or other preliminary research. Because PCK develops primarily through practical experience and reflection on it (van Driel and Berry, 2020), instruments designed for novices (i.e., undergraduates) need to be rather simple. In contrast, instruments for experts (i.e., experienced teachers) need to be more difficult. Furthermore, target groups with larger variability among their responses require a higher number of items in order to achieve satisfactory discrimination and reliability indices. Finally, because of the novices’ tendency toward *Surface Thinking*, formal task characteristics should be kept constant across items or varied systematically to avoid or control for construct-irrelevant sources of variance.

### 5.6. Limitations and future work

The main limitation of our study concerns the sample, both its size and its composition. On the one hand, the findings for RQ2 indicate that the overall sample was too small to detect relevant effects of medium size. Correspondingly, the power was only less than 0.30. Conversely, to achieve a satisfactory power of 0.80, the two groups would have had to include at least *n* = 50 participants each. However, due to the large time investment required to conduct the study and, in particular, to code the interviews, it was not possible to include more participants. On the other hand, the results for RQ3, precisely the implausibly high effect sizes of more than 2 and 3 standard deviations, respectively, suggest that the two groups were each too homogeneous. We expect these group differences to diminish with increasing sample size and larger heterogeneity of participants. Against this background, our results are not generalizable in their present form. Nevertheless, our study has provided important initial evidence that may be useful in the construction of test instruments to assess PCK.

A second limitation concerns the overall generalizability of our results. Even if we assume that our findings might be useful in constructing other PCK tests, we can (of course) not guarantee that they are equally relevant to tasks other than scientific reasoning due to the topic-specificity of the PCK construct itself.

In the next step, we will evaluate the instrument based on the feedback from the experts. In addition, the validity of the test score interpretation will be examined considering sources of validity evidence.

## Data availability statement

The raw data supporting the conclusions of this article will be made available by the authors, without undue reservation.

## Ethics statement

The studies involving humans were approved by Leibniz Institute for Science and Mathematics Education Ethics Committee. The studies were conducted in accordance with the local legislation and institutional requirements. The participants provided their written informed consent to participate in this study.

## Author contributions

LG and MK initially developed the multiple-choice instrument used in this study. TL further developed the instrument, conducted the interviews, developed the category system, analyzed and interpreted the data, and has written large parts of the manuscript. VW helped with the analysis and interpretation of the data. VW, LG, and MK were major contributors in writing the manuscript. MK supervised the entire process. All authors read and approved the final manuscript.

## References

[ref1] Alonzo A. C. Berry A. Nilsson P. (2019). “Unpacking the complexity of science teachers’ PCK in action: enacted and personal PCK” in Repositioning pedagogical content knowledge in teachers’ knowledge for teaching science. eds. Hume A. Cooper R. Borowski A. (Singapore: Springer Singapore), 271–286.

[ref2] Alonzo A. C. Kobarg M. Seidel T. (2012). Pedagogical content knowledge as reflected in teacher-student interactions: analysis of two video cases. J. Res. Sci. Teach. 49, 1211–1239. doi: 10.1002/tea.21055

[ref3] American Educational Research Association (AERA), American Psychological Association (APA) and National Council on Measurement in Education (NCME). (2014). Standards for educational and psychological testing. Washington, DC: American Educational Research Association.

[ref4] Australian Curriculum, Assessment and Reporting Authority (ACARA). (2022). Science: F-10 Version 9.0. Curiculum content 7–10. Available at: https://v9.australiancurriculum.edu.au/f-10-curriculum/f-10-curriculum-overview (Accessed June 10, 2023).

[ref5] Baumert J. Kunter M. (2013). “The COACTIV model of teachers’ professional competence” in Mathematics teacher education. Cognitive activation in the mathematics classroom and professional competence of teachers: Results from the COACTIV project. eds. Kunter M. Baumert J. Blum W. Klusmann U. Krauss S. Neubrand M., vol. 8 (New York: Springer), 25–38.

[ref6] Baumert J. Kunter M. Blum W. Brunner M. Voss T. Jordan A.. (2010). Teachers’ mathematical knowledge, cognitive activation in the classroom, and student progress. Am. Educ. Res. J. 47, 133–180. doi: 10.3102/0002831209345157

[ref7] Blömeke S. Jentsch A. Ross N. Kaiser G. König J. (2022). Opening up the black box: teacher competence, instructional quality, and students’ learning progress. Learn. Instr. 79:101600. doi: 10.1016/j.learninstruc.2022.101600

[ref8] British Columbia Teachers’ Council (BCTC). (2019). Professional standards for BC educators. Available at: https://www2.gov.bc.ca/assets/gov/education/kindergarten-to-grade-12/teach/teacher-regulation/standards-for-educators/edu_standards.pdf (Accessed June 10, 2023).

[ref9] Carlson J. Daehler K. R. (2019). “The refined consensus model of pedagogical content knowledge in science education” in Repositioning pedagogical content knowledge in teachers’ knowledge for teaching science. eds. Hume A. Cooper R. Borowski A. (Singapore: Springer Singapore), 77–92.

[ref10] Chan K. K. H. Hume A. (2019). “Towards a consensus model: literature review of how science teachers’ pedagogical content knowledge is investigated in empirical studies” in Repositioning pedagogical content knowledge in teachers’ knowledge for teaching science. eds. Hume A. Cooper R. Borowski A. (Singapore: Springer Singapore), 3–76.

[ref11] Cobern W. W. Schuster D. Adams B. Skjold B. A. Muğaloğlu E. Z. Bentz A.. (2014). Pedagogy of science teaching tests: formative assessments of science teaching orientations. Int. J. Sci. Educ. 36, 2265–2288. doi: 10.1080/09500693.2014.918672

[ref12] Cohen J. (1988). Statistical power analysis for the behavioral sciences. 2nd Edn. New York: Taylor and Francis.

[ref001] Clough E. E. Driver R. (1986). A study of consistency in the use of students’ conceptual frameworks across different task contexts. Sci. Educ. 70, 473–496. doi: 10.1002/sce.3730700412

[ref13] De Groot A. D. (1965). Thought and choice in chess. Hawthorne, NY: Mouton publishers.

[ref14] Dresing T. Pehl T. (2018). Praxisbuch Interview, Transkription & Analyse: Anleitungen und Regelsysteme für qualitativ Forschende [interview, transcription, & analysis practice book: instructions and rule systems for qualitative researchers]. 8th Edn. Marburg.

[ref15] Ercikan K. Pellegrino J. W. (2017). “Validation of score meaning using examinee response processes for the next generation of assessments,” in Validation of score meaning for the next generation of assessments: The use of response processes. eds. Ercikan K. Pellegrino J. W. (New York: Routledge), 1–8.

[ref16] Ericsson K. A. (2006). “The influence of experience and deliberate practice on the development of superior expert performance,” in The Cambridge handbook of expertise and expert performance. eds. Ericsson K. A. Charness N. Feltovich P. J. Hoffman R. R. (Cambridge: Cambridge University Press).

[ref17] Forsythe M. E. Criswell B. A. Arias A. M. Ellis J. A. Escalada L. Johnson H. J.. (2022). The framework for analyzing video in science teacher education (FAVSTE). J. Sci. Teach. Educ. 33, 621–640. doi: 10.1080/1046560X.2021.1970698

[ref18] Gess-Newsome J. (2015). “A model of teacher professional knowledge and skill inklunding PCK” in Teaching and learning in science series. Re-examining pedagogical content knowledge in science education. eds. Berry A. Friedrichsen P. Loughran J.. 1st ed (New York, NY: Routledge), 28–42.

[ref19] Göhner M. Krell M. (2022). Preservice science teachers’ strategies in scientific reasoning: the case of modeling. Res. Sci. Educ. 52, 395–414. doi: 10.1007/s11165-020-09945-7

[ref20] Großmann L. Krüger D. (2023). “Identifying performance levels of enacted pedagogical content knowledge in trainee biology teachers’ lesson plans” in Fostering scientific citizenship in an uncertain world. Contributions from science education research. eds. Carvalho G. S. Afonso A. S. Anastácio Z., vol. 13 (New York: Springer International Publishing), 95–116.

[ref21] Großschedl J. Welter V. Harms U. (2019). A new instrument for measuring pre-service biology teachers’ pedagogical content knowledge: the PCK-IBI. J. Res. Sci. Teach. 56, 402–439. doi: 10.1002/tea.21482

[ref22] Günther S. L. Fleige J. Belzen A. U. Krüger D. (2019). Using the case method to foster preservice biology teachers’ content knowledge and pedagogical content knowledge related to models and modeling. J. Sci. Teach. Educ. 30, 321–343. doi: 10.1080/1046560X.2018.1560208

[ref23] Haladyna T. M. (2004). Developing and validating multiple-choice test items 3rd Edn London: Routledge Taylor & Francis Group.

[ref24] Hattie J. (2008). Visible learning: A synthesis of over 800 meta-analyses relating to achievement. New York: Routledge.

[ref25] Hogan T. Rabinowitz M. Craven J. A. (2003). Representation in teaching: inferences from research of expert and novice teachers. Educ. Psychol. 38, 235–247. doi: 10.1207/S15326985EP3804_3

[ref26] Hubley A. M. Zumbo B. D. (2017). “Response processes in the context of validity: setting the stage” in Understanding and investigating response processes in validation research. eds. Zumbo B. D. Hubley A. M., vol. 69 (New York: Springer International Publishing), 1–12.

[ref27] Jin H. Shin H. Johnson M. E. Kim J. Anderson C. W. (2015). Developing learning progression-based teacher knowledge measures. J. Res. Sci. Teach. 52, 1269–1295. doi: 10.1002/tea.21243

[ref28] Justi R. van Driel J. (2006). The use of the interconnected model of teacher professional growth for understanding the development of science teachers’ knowledge on models and modelling. Teach. Teach. Educ. 22, 437–450. doi: 10.1016/j.tate.2005.11.011

[ref29] Jüttner M. Boone W. Park S. Neuhaus B. J. (2013). Development and use of a test instrument to measure biology teachers’ content knowledge (CK) and pedagogical content knowledge (PCK). Educ. Assess. Eval. Account. 25, 45–67. doi: 10.1007/s11092-013-9157-y

[ref30] Kane M. T. Mislevy R. (2017). “Validating score interpretations based on response processes” in Validation of score meaning for the next generation of assessments: The use of response processes. eds. Ercikan K. Pellegrino J. W. (New York: Routledge), 11–24.

[ref31] Karal I. S. Alev N. (2016). Development of pre-service physics teachers’ pedagogical content knowledge (PCK) throughout their initial training. Teach. Dev. 20, 162–180. doi: 10.1080/13664530.2015.1124138

[ref32] Keller M. M. Neumann K. Fischer H. E. (2017). The impact of physics teachers’ pedagogical content knowledge and motivation on students’ achievement and interest. J. Res. Sci. Teach. 54, 586–614. doi: 10.1002/tea.21378

[ref33] Khan S. Krell M. (2019). Scientific reasoning competencies: a case of preservice teacher education. Can. J. Sci. Math. Technol. Educ. 19, 446–464. doi: 10.1007/s42330-019-00063-9

[ref34] Kind V. Chan K. K. H. (2019). Resolving the amalgam: connecting pedagogical content knowledge, content knowledge and pedagogical knowledge. Int. J. Sci. Educ. 41, 964–978. doi: 10.1080/09500693.2019.1584931

[ref35] Kleickmann T. Richter D. Kunter M. Elsner J. Besser M. Krauss S.. (2013). Teachers’ content knowledge and pedagogical content knowledge. J. Teach. Educ. 64, 90–106. doi: 10.1177/0022487112460398

[ref36] Kline P. (2015). A handbook of test construction (psychology revivals) Introduction to Psychometric Design. New York: Routledge.

[ref40] Knuuttila T. (2011). Modelling and representing: an artefactual approach to model-based representation. Stud. Hist. Phil. Sci. 42, 262–271. doi: 10.1016/j.shpsa.2010.11.034

[ref41] König J. Hanke P. Glutsch N. Jäger-Biela D. Pohl T. Becker-Mrotzek M.. (2022). Teachers’ professional knowledge for teaching early literacy: conceptualization, measurement, and validation. Educ. Assess. Eval. Account. 34, 483–507. doi: 10.1007/s11092-022-09393-z

[ref42] Kozma R. B. Russell J. (1997). Multimedia and understanding: expert and novice responses to different representations of chemical phenomena. J. Res. Sci. Teach. 34, 949–968. doi: 10.1002/(SICI)1098-2736(199711)34:9<949::AID-TEA7>3.0.CO;2-U

[ref43] Krell M. (2018). Schwierigkeitserzeugende Aufgabenmerkmale bei multiple-choice-Aufgaben zur Experimentierkompetenz im Biologieunterricht: Eine Replikationsstudie [difficulty-generating item characteristics in multiple-choice items for experimental literacy in biology education: a replication study]. Zeitschrift für Didaktik der Naturwissenschaften 24, 1–15. doi: 10.1007/s40573-017-0069-0

[ref44] Krell M. Vorholzer A. Nehring A. (2022). Scientific reasoning in science education: from global measures to fine-grained descriptions of students’ competencies. Educ. Sci. 12:97. doi: 10.3390/educsci12020097

[ref45] Krepf M. Plöger W. Scholl D. Seifert A. (2018). Pedagogical content knowledge of experts and novices-what knowledge do they activate when analyzing science lessons? J. Res. Sci. Teach. 55, 44–67. doi: 10.1002/tea.21410

[ref46] Kunter M. Klusmann U. Baumert J. Richter D. Voss T. Hachfeld A. (2013). Professional competence of teachers: effects on instructional quality and student development. J. Educ. Psychol. 105, 805–820. doi: 10.1037/a0032583

[ref47] Kunter M. Trautwein U. (2013). Psychologie des Unterrichts [psychology of teaching]. Weinheim: Beltz.

[ref48] Landis J. R. Koch G. G. (1977). The measurement of bbserver agreement for categorical data. Biometrics 33:159. doi: 10.2307/2529310 843571

[ref002] Lenhard W. Lenhard A. (2016). Computation of effect sizes. Available at: https://www.psychometrica.de/effektstaerke.html (Accessed June 10, 2023).

[ref49] Lieberei T. Großmann L. Welter V. D. E. Krüger D. Krell M. (2023). Entwicklung und Evaluation eines Instruments zur Erhebung fachdidaktischen Wissens angehender Biologielehrkräfte im Kompetenzbereich Erkenntnisgewinnung: PCKSR-bio [Development and evaluation of an instrument to assess pedagogical content knowledge about scientific reasoning: PCKSR-bio]. In Herausforderung Zukunft: Internationale Tagung der Fachsektion Didaktik der Biologie (FDdB) im VBIO [International conference oft he FDdB]. eds. Müller S. Schaal S. (Lüneburg: Pädagogische Hochschule Lüneburg), 491–493.

[ref50] Loughran J. Mulhall P. Berry A. (2004). In search of pedagogical content knowledge in science: developing ways of articulating and documenting professional practice. J. Res. Sci. Teach. 41, 370–391. doi: 10.1002/tea.20007

[ref51] Magnusson S. Krajcik J. Borko H. (1999). “Nature, sources, and development of pedagogical content knowledge for science teaching” in Examining pedagogical content knowledge. eds. Gess-Newsome J. Lederman N. G. (Alphen aan den Rijn: Kluwer Academic Publishers), 95–132.

[ref52] Mahler D. Großschedl J. Harms U. (2017). Using doubly latent multilevel analysis to elucidate relationships between science teachers’ professional knowledge and students’ performance. Int. J. Sci. Educ. 39, 213–237. doi: 10.1080/09500693.2016.1276641

[ref53] Martinez M. E. (1999). Cognition and the question of test item format. Educ. Psychol. 34, 207–218. doi: 10.1207/s15326985ep3404_2

[ref54] Mathesius S. Hartmann S. Upmeierzu Belzen A. Krüger D. (2016). “Scientific reasoning as an aspect of preservice biology teacher education,” in The future of biology education research. Proceedings of the 10th conference of European researchers in didactics of biology (ERIDOB). eds. Tal T. Yarden A. (Haifa: Technion), 93–110.

[ref55] Messick S. (1996). “Validity of performance assessments,” in Technical issues in large-scale performance assessment. ed. Phillips G. W. (Washington, D.C.: National Center for Education Statistics), 1–18.

[ref56] Ministry of Education (2008a). The Ontario curriculum: Grades 11 and 12. Science. Available at: https://www.edu.gov.on.ca/eng/curriculum/secondary/2009science11_12.pdf (Accessed June 10, 2023).

[ref57] Ministry of Education. (2008b). The Ontario curriculum: Grades 9 and 10. Science. Available at: https://www.edu.gov.on.ca/eng/curriculum/secondary/science910_2008.pdf (Accessed June 10, 2023).

[ref58] Ministerio de Educación (MINEDUC). (2012). Estándares orientadores para carreras de Pedagogía en Educación Media [Guiding Standards for Secondary Education Pedagogy Careers]. Available at: https://www.cpeip.cl/wp-content/uploads/2018/09/Est%C3%A1ndares_Media.pdf

[ref59] Ministerio de Educación (MINEDUC). (2015). Bases Curriculares: 7° básico a 2° medio [curricular basics: 7th basic to 2nd middle grade]. Available at: https://www.curriculumnacional.cl/614/articles-34949_Bases.pdf

[ref60] Ministerio de Educación (MINEDUC). (2019). Bases Curriculares: 3° y 4° medio [curricular bases: 3rd and 4th grades]. Available at: https://www.curriculumnacional.cl/614/articles-91414_bases.pdf

[ref004] Nehm R. H. Ridgway J. (2011). What do experts and novices “see” in evolutionary problems? Evol.: Educ. 4, 666–679. doi: 10.1007/s12052-011-0369-7

[ref61] Neumann K. Kind V. Harms U. (2019). Probing the amalgam: the relationship between science teachers’ content, pedagogical and pedagogical content knowledge. Int. J. Sci. Educ. 41, 847–861. doi: 10.1080/09500693.2018.1497217

[ref62] New South Wales (NSW) Education Standards Authority. (2018). Australian professional standards for teachers: Teacher accreditation. Available at: https://educationstandards.nsw.edu.au/wps/wcm/connect/9ba4a706-221f-413c-843b-d5f390c2109f/australian-professional-standards-teachers.pdf?MOD=AJPERES (Accessed June 10, 2023).

[ref63] O’Connor C. Joffe H. (2020). Intercoder reliability in qualitative research: debates and practical guidelines. Int. J. Qual. Methods 19, 160940691989922–160940691989913. doi: 10.1177/1609406919899220

[ref005] Opfer J. E. Nehm R. H. Ha M. (2012). Cognitive foundations for science assessment design: Knowing what students know about evolution. J. Res. Sci. Teach. 49, 744–777. doi: 10.1002/tea.21028

[ref64] Osborne J. (2013). The 21st century challenge for science education: assessing scientific reasoning. Think. Skills Creat. 10, 265–279. doi: 10.1016/j.tsc.2013.07.006

[ref65] Park S. Chen Y.-C. (2012). Mapping out the integration of the components of pedagogical content knowledge (PCK): examples from high school biology classrooms. J. Res. Sci. Teach. 49, 922–941. doi: 10.1002/tea.21022

[ref66] Park S. Oliver J. S. (2008). Revisiting the conceptualisation of pedagogical content knowledge (PCK): PCK as a conceptual tool to understand teachers as professionals. Res. Sci. Educ. 38, 261–284. doi: 10.1007/s11165-007-9049-6

[ref67] Park S. Suh J. Seo K. (2018). Development and validation of measures of secondary science teachers’ PCK for teaching photosynthesis. Res. Sci. Educ. 48, 549–573. doi: 10.1007/s11165-016-9578-y

[ref68] Rodriguez M. C. (2005). Three options are optimal for multiple-choice items: a meta-analysis of 80 years of research. Educ. Meas. Issues Pract. 24, 3–13. doi: 10.1111/j.1745-3992.2005.00006.x

[ref69] Rönnebeck S. Bernholt S. Ropohl M. (2016). Searching for a common ground – a literature review of empirical research on scientific inquiry activities. Stud. Sci. Educ. 52, 161–197. doi: 10.1080/03057267.2016.1206351

[ref70] Sadler P. M. Sonnert G. Coyle H. P. Cook-Smith N. Miller J. L. (2013). The influence of teachers’ knowledge on student learning in middle school physical science classrooms. Am. Educ. Res. J. 50, 1020–1049. doi: 10.3102/0002831213477680

[ref71] Sannert R. Krell M. (2023). A professional development program to foster science teachers’ professional competence, enhance classroom practice, and improve student outcomes related to scientific reasoning. Progress Sci. Educ. 6, 47–62. doi: 10.25321/PRISE.2023.1401

[ref72] Schmitt A. K. (2016). Entwicklung und evaluation einer Chemielehrerfortbildung zum Kompetenzbereich Erkenntnisgewinnung [development and evalution on a chemistry teacher training course on scientific reasoning]. Logos Verlag Berlin.

[ref007] Schnotz W. Baadte C. (2015). Surface and deep structures in graphics comprehension. Mem. Cogn. 43, 605–618. doi: 10.3758/s13421-014-0490-2 25465898

[ref73] Schreier M. (2012). Qualitative content analysis in practice. Thousand Oaks, CA: SAGE.

[ref74] Schreier M. (2014). Varianten qualitativer Inhaltsanalyse: Ein Wegweiser im Dickicht der Begrifflichkeiten [Ways of doing qualitative content analysis: disentangling terms and terminologies]. Forum Qualitative Sozialforschung 15:18. doi: 10.17169/fqs-15.1.2043

[ref75] Schuster D. Cobern W. Applegate B. Schwartz R. Vellom P. Undreiu A. (2006). “Assessing pedagogical content knowledge of inquiry science teaching: Developing an assessment instrument to support the undergraduate preparation of elementary teachers to teach science as inquiry” in STEM Assessment Conference. eds. Deeds D. Callen B. (Washington, DC)

[ref76] Schweizer K. DiStefano C. (2016). “Principles and methods of test construction: standards and recent advances” in Psychological assessment–science and practice. 1st ed (Göttingen: Hogrefe publishing)

[ref37] Sekretariat der Ständigen Konferenz der Kultusminister der Länder in der Bundesrepublik Deutschland (KMK). (2005). Bildungsstandards im Fach Biologie für den Mittleren Schulabschluss Beschluss vom 16.12.2004. [educational standards in the subject biology for the intermediate school leaving. Resolution of 16.12.2004]. Available at: https://www.kmk.org/fileadmin/veroeffentlichungen_beschluesse/2004/2004_12_16-Bildungsstandards-Biologie.pdf (Accessed June 10, 2023).

[ref38] Sekretariat der Ständigen Konferenz der Kultusminister der Länder in der Bundesrepublik Deutschland (KMK). (2019). Ländergemeinsame inhaltliche Anforderungen für die Fachwissenschaften und Fachdidaktiken in der Lehrerbildung [common content-specific requirements oft he federal states for the subject sciences and subject didactics in teacher education]. Available at: https://www.kmk.org/fileadmin/Dateien/veroeffentlichungen_beschluesse/2008/2008_10_16-Fachprofile-Lehrerbildung.pdf (Accessed June 10, 2023).

[ref39] Sekretariat der Ständigen Konferenz der Kultusminister der Länder in der Bundesrepublik Deutschland (KMK). (2020). Bildungsstandards im Fach Biologie für die Allgemeine Hochschulreife [educational tandards in the subject biology for the general higher education entrance qualification]. Available at: https://www.kmk.org/fileadmin/Dateien/veroeffentlichungen_beschluesse/2020/2020_06_18-BildungsstandardsAHR_Biologie.pdf (Accessed June 10, 2023).

[ref78] She J. Chan K. K. H. (2022). Situated and dynamic versus declarative and static forms of pedagogical content knowledge: an evaluation of the differences in test reactions and performance. J. Res. Sci. Teach. 60, 568–607. doi: 10.1002/tea.21810

[ref79] Shulman L. S. (1986). Those who understand: knowledge growth in teaching. Educ. Res. 15, 4–14. doi: 10.3102/0013189X015002004

[ref80] Shulman L. S. (1987). Knowledge and teaching: foundations of the new reform. Harv. Educ. Rev. 57, 1–23. doi: 10.17763/haer.57.1.j463w79r56455411

[ref81] Smith P. S. Banilower E. R. (2015). “Assessing PCK: a new application of the uncertainty principle” in Teaching and learning in science series. Re-examining pedagogical content knowledge in science education. eds. Berry A. Friedrichsen P. Loughran J.. 1st ed (New York: Routledge), 88–103.

[ref82] Sorge S. Kröger J. Petersen S. Neumann K. (2019a). Structure and development of pre-service physics teachers’ professional knowledge. Int. J. Sci. Educ. 41, 862–889. doi: 10.1080/09500693.2017.1346326

[ref83] Sorge S. Stender A. Neumann K. (2019b). “The development of science teachers’ professional competence” in Repositioning pedagogical content knowledge in teachers’ knowledge for teaching science. eds. Hume A. Cooper R. Borowski A. (Singapore: Springer Singapore), 151–166.

[ref84] Tepner O. Dollny S. (2014). “Entwicklung eines Testverfahrens zur analyse fachdidaktischen Wissens [development of a test procedure for the analysis of pedagogical content knowledge]” in Methoden in der naturwissenschaftsdidaktischen Forschung [methods in science educational research]. eds. Krüger D. Parchmann I. Schecker H. (Berlin: Springer Spektrum), 311–323.

[ref85] Upmeierzu Belzen A. van Driel J. Krüger D. (2019). Introducing a framework for modeling competence. In Upmeierzu Belzen A Krüger D. Driel J. van (Eds.), Towards a competence-based view on models and modeling in science education (Vol. 12, pp. 3–19). New York: Springer International Publishing.

[ref86] Van Driel J. Berry A. (2020). “Pedagogical content knowledge in preservice teacher education” in Encyclopedia of teacher education. ed. Peters M. A. (Singapore: Springer Nature Singapore)

[ref87] Wirtz M. A. Caspar F. (2002). Beurteilerübereinstimmung und Beurteilerreliabilität: Methoden zur Bestimmung und Verbesserung der Zuverlässigkeit von Einschätzungen mittels Kategoriensystemen und Ratingskalen [Intercoder aggreement and intercoder reliability]. Göttingen: Hogrefe Verl. für Psychologie.

[ref88] Wolff C. E. van den Bogert N. Jarodzka H. Boshuizen H. P. A. (2015). Keeping an eye on learning: differences between expert and novice teachers’ representations of classroom management events. J. Teach. Educ. 66, 68–85. doi: 10.1177/0022487114549810

