# Supplementary material for: Findings from the expert-novice paradigm on differential response behavior among multiple-choice items of a pedagogical content knowledge test – implications for test development
Source: Front Psychol. 2023 Oct 18;14:1240120. doi: 10.3389/fpsyg.2023.1240120 (PMC10619162; doi:10.3389/fpsyg.2023.1240120)
Supplement: Supplementary file 1 [file Table_1.pdf]

Supplementary Material

Findings from the Expert-Novice Paradigm on Differential Response Behavior among Multiple-Choice Items of a PCK Test - Implications for Test Development

Tobias Lieberei*, Virginia Deborah Elaine Welter, Leroy Großmann, Moritz Krell

*** Correspondence:** Corresponding Author: lieberei@leibniz-ipn.de

Supplementary Table 1

Full version of the coding system

Categories		Description	Empirical Evidence
Revision Note		Justifications of novices and experts that indicate a revision of the items. These can be explicit (i.e., addressed directly) or implicit (i.e., it can be seen that the item was not understood).	
Answer			
Right Answer		The item is answered correctly, only the final explanation and choice counts.	
Wrong Answer		The item is answered incorrectly.	
No Answer		No answer is given to the item.	
Explanation			
Intended		The explanation of the choice of an answer option is as intended (i.e., as intended when the item was created).	
Not Intended		The explanation of the choice of an answer option is not intended (i.e., not as intended when the item was created).	
Surface Thinking			
Item Stem	Student Characteristics	The (as inadequate considered) description of the learning group is used for the explanation.<br>Only surface characteristics of the learning group are mentioned, such as the grade level, the expected competencies (not based on a subject didactic assessment, but without further explanations). …	*I think that the phrase 'target-oriented approach' sounds a little too high for eighth graders [and is therefore not used by them]* - (Novice 10)

Categories		Description	Empirical Evidence
		The language used in students' statements mentioned in the item stem is used to explain the choice of answer options. Answer options in everyday language are chosen only for this reason, answer options in technical language are excluded only for this reason.<br><br>Based on Kunter and Trautwein (2013).	
	Topic	The topic of the classroom situation addressed in the stem is used to justify the chosen answer option without using PCK.<br><br>Based on Kunter and Trautwein (2013).	*[The students know that they can change the CO2 concentration by adding sparkling water, because] I simply believe that the students know the difference between tap water and sparkling water in the eighth grade […]. Nowadays I think they know the difference, in times of SodaStream and things like that.* - (Novice 3)
	Teaching Methods and Social Forms	The justification of the choice of answer options is made with reference to the teaching methods or social forms addressed in the classroom situation <u>without using PCK</u>.<br>The answer option is selected because the teaching situation in the item is too easy/difficult for the students (<u>without using PCK</u>).<br><br>Based on Kunter and Trautwein (2013).	*I didn't choose answer option (1) because I don't think that DNA is a good topic where you can start with students making their own models themselves.* - (Novice 3)<br><br>*[I think the students are more likely to develop this idea] because they work in different groups and really have a lot of [different] results and by comparing all them they can develop the rule themselves […].* - (Novice 8)
Personal Experience		The selection is based on personal experience. It includes both reported experiences from the perspectives of student and teacher. Only coded if own experiences are explicit and specifically mentioned.	*[...] I can only say that based on my personal experience, [...] I don't think that I would have understood anything about biology as a science in eighth grade, […] that models are not only being developed for students, but*

Categories	Description	Empirical Evidence
		also for people who really know about it. - (Novice 3)
Intuition	The selection is explained intuitively. It is argued with the feeling (e.g., “I think this is right”), *without using PCK*.<br>The participant cannot explain in more detail why the choice was made. The reason given is that an item/answer option is not understood.<br><br>The rationale for selecting the response option is not from a constructivist perspective, but from a transmissive perspective on teaching and learning (Seidel & Reiss, 2014).	*I would take [this] answer option because [I think the students would say this because] I don’t know. So somehow- I don’t know, I think that’s how they phrase it, I don’t know* - (Novice 4)<br><br>*[…] it’s important that the students have an instruction, because [as a teacher] you should support them in experimenting.* - (Novice 1)
Wrong Interpretation	The necessary understanding exists but the item / answer option is interpreted differently than intended. As a result, the choice of answer option and / or the explanation is incorrect.<br>Serves as an indication that the phrasing of the item is unclear, which is why it is often coded with *“Revision Note”*.	*So they have already used models frequently. From this, you could conclude that the models they have used before were manufactured models […].* - (Expert 1)
Misconception	The reason for selection is wrong/missing knowledge (e.g., in a task on modelling skills, the person has no knowledge that these can be used to gain knowledge).	*[I didn’t choose the answer option] "Biological models are created by scientists to conduct research with them." […] because you are not researching with models, but the actual things. Well, in science you don’t do research with models, but models more or less reflect what is being researched in science […].* - (Novice 6)
Pedagogical Knowledge	Explanations are not based on PCK, but on general pedagogical ideas or argued with pedagogical beliefs.<br>The expected teaching time for a certain action is used to justify the choice of response options.<br>Constructivist justifications are used without reference to PCK (e.g., “I choose this	*I wouldn’t conduct different experiments [in the lesson], because I think that would be a bit too much for one lesson. I think one experiment is enough […].* - (Novice 1)

Categories	Description	Empirical Evidence
	because they work there themselves and thus they learn better”).<br><br>Distincton based on Shulman (1986, 1987) and the empirical evidence by Krepf et al. (2018).	*Well, for example, boys learn somehow also often better if they touch something or make something themselves with their hands. I think that modelling is much more understandable if you rebuilt the models yourself* - (Novice 7)
Content Knowledge	Explanation of the selection is based on CK, no reference to PCK.<br><br>Distincton based on Shulman (1986, 1987) and the emprirical evidence by Krepf et al. (2018).	*I wouldn’t choose this because models usually do not completely [represent the reality] […]. A model does not show exactly how something is in reality, but only approximately so that you can imagine it better.* - (Novice 8)
No Relation to Scientific Reasoning	Explanations of the selection is based on other reasons for the decision (e.g., what teachers or students would answer).<br>The choice is explained by the exclusion of other response options.<br>The linguistic wording of the response option is used as an explanation for non-selection.<br>Arguments are made with other competences that cannot be assigned to scientific reasoning (e.g., model criticism in the competence area modelling).	*[…] That's why I think that the model critique is much more conducive to promoting an appropriate scientific understanding in the students than the other answer.* - (Novice 3)
Deep Thinking		
Orientations to Teaching Science	Choice of an answer option is explained with purposes and goals of science teaching with reference to *pedagogical content knowledge*. Explanations regarding beliefs about (biology) teaching in general are not included. (Park & Chen, 2012; Park & Oliver, 2008)<br><br>Explanations are based on competence models or other theoretical models (e.g., *SDDS model*).	*[I] would probably chose this answer option because that’s most likely to encompass the meaningfulness of experiments. If you think about the SDDS model, and the competencies described there. For example, analysis of evidence.* - (Expert 8)
Knowledge of Students’ Understanding	Choice of an answer option is based on knowledge about student perceptions (i.e., learning difficulties, motivation and differences in abilities, individual learning styles and interests) as well as knowledge about common conceptions and the prior	*[These two answer options are implausible] […] because students tend to see models as visualizations rather than for research […].*

Categories		Description	Empirical Evidence
		knowledge of students (Park & Chen, 2012; Park & Oliver, 2008).	*[The other answer option is more suitable] because it addresses the reality of the students'. This is because it is the function of models that they know and that they can reproduce.* - (Expert 1)
Knowledge of Science Curriculum		Choice of an answer option is explained with the use of the curriculum; argumentation on a horizontal or vertical level; explanation of the choice based on the curriculum as a whole. Core conceptions of the curriculum are applied to the item situation. Referencing directly to the curriculum (Park & Chen, 2012; Park & Oliver, 2008).	*[It reminds me of] the curriculum biology secondary level 1 [opens framework on the computer and searches in]. So then we have "dealing with models". There we have the three big areas "Use", "Test" and "Change". And I think these answers could be assigned quite well.* - (Expert 2)
Knowledge of Instructional Strategies and Representations of Teaching Science	Topic-Specific Strategies	Choice of an answer option is explained based on teaching strategies related to the specific topic in the item situation. Thereby, the focus of the explanation is on teaching methods relating to a specific topic, not teaching strategies in general (Park & Oliver, 2008).	*But to think about this example with this specific subject content, I would not reflect on the goal-oriented procedure of hypothesis formation [for this topic]*. - (Expert 2)
	Subject-Specific Strategies	Choice of an answer option is explained based on teaching strategies relating to achieve the goals of biology teaching related to scientific methods and scientific reasoning. Includes also inquiry-based teaching strategies (Park & Chen, 2012; Park & Oliver, 2008).	*I would exclude this answer, because it is not about developing competencies about experimentation, but only about finding a result to the research question.* - (Expert 8)
Knowledge of Assessment of Science Learnings		Choice of an answer option is explained based on "knowledge of specific instruments, approaches, or activities" (p. 266) for the assessment of science learnings. (Park & Oliver, 2008)	No Coding
